# Supplementary material for: Dirac-source diode with sub-unity ideality factor
Source: Nat Commun. 2022 Jul 26;13:4328. doi: 10.1038/s41467-022-31849-5 (PMC9325700; doi:10.1038/s41467-022-31849-5)
Supplement: Supplementary file 1 — supplementary information [file 41467_2022_31849_MOESM1_ESM.pdf]

# Supplementary Information

## Dirac-source Diode with Sub-unity Ideality Factor

Gyuho Myeong[1†], Wongil Shin[1†], Kyunghwan Sung[1†], Seungho Kim[1], Hongsik Lim[1], Boram Kim[1], Taehyeok Jin[1], Jihoon Park[1], Taehun Lee[1], Michael S. Fuhrer[2], Kenji Watanabe[3], Takashi Taniguchi[3], Fei Liu[4, 5**], Sungjae Cho[1*]

[1] Department of Physics, Korea Advanced Institute of Science and Technology (KAIST), Daejeon, Korea

[2] ARC Centre of Excellence in Future Low-Energy Electronics Technologies, and School of Physics and Astronomy, Monash University, Clayton, Victoria 3800, Australia

[3] National Institute for Materials Science, Namiki Tsukuba Ibaraki 305-0044, Japan

[4] School of Integrated Circuits, Peking University, Beijing, 100871, China
[5] Beijing Advanced Innovation Center for Integrated Circuits, Beijing, 100871, China

[†]These authors contributed equally to this work.
* Corresponding author, S. C, Email: sungjae.cho@kaist.ac.kr

** Corresponding author, F. L, Email: feiliu@pku.edu.cn

## Supplementary Texts

### 1. Electron density distribution of normal metallic and Dirac sources

The continuous density of states (DOS) of a normal metallic source exhibits the Boltzmann distributed electron density given by $n(E) \approx \exp((E_F - E)/k_BT)$. For a Dirac source, the linearly varied DOS produces a super-exponentially decreasing electron density (given by $n(E) \approx (E_{Dirac} - E) \exp((E_F - E)/k_BT)$) with decreasing energy. Supplementary Figure 1 compares the electron densities of the normal metallic and Dirac sources. The green dotted curve in Supplementary Figure 1b indicates the electron density of the normal metallic source to facilitate its comparison against that of the Dirac source. Therefore, carrier distribution in the source of DS diode and FET has steeper superexpontial decays with energy, overcoming thermionic exponential distribution of carrier density in conventional diode and FET. Therefore, the ideality factor and subthreshold swing of our DS diode and DS FET can have lower value than the conventional limits (ideality factor = 1 and subthreshold swing = 60mV/dec at 300K), which again originates from thermionic carrier distribution of the source in the conventional diode and FET.

### 2. Electron doping of $MoS_2$ channel

To check the doping level of $MoS_2$ channel, we measured gate dependence of $I_D$, shown in Supplementary Figure 3. As shown in Supplementary Figure 3a, we measured gate dependence of $MoS_2$ channel with two-probe measurement. In Supplementary Figure 3b, the channel showed n-type transfer characteristic at zero gate voltage.

### 3. Contact quality between normal and two-dimensional (2D) metals

With regard to the fabrication of electronic devices using transition metal dichalcogenides (TMD) materials, several recent studies have reported the improvement of the metal–semiconductor interface as a means for enhancing device performance. For example, the use of the 2D-semimetal, pre-patterned metal contacts, as well as the method of releasing evaporated metal on a TMD substrate have been suggested as ways to improve the metal–semiconductor interface[1-3]. These methods are intended to prevent the damage caused to the TMD material during the direct metal deposition. Supplementary Figure 4 illustrates the

schematic process of establishing the metal-evaporation and 2D-metal contacts. The metal-deposition process comprises three steps: (1) coating polymethyl methacrylate (PMMA) resist, pattern lithography and development, (2) metal deposition, and (3) lift-off. Each step is influenced by a factor that limits the realizable contact and device quality. During lithography pattern development, the region of metal deposition is exposed to various chemicals (resistive coating, developer, and stopper) and air. During metal deposition, hot metal vapor adheres to the contact region, thereby causing structural deformations within the TMD material[1]. During lift-off, the channel region of the device is exposed to a solvent. Thus, electronic devices fabricated using direct metal deposition are subjected to the occurrence of chemical contamination and structural deformation in both the metal-contact and channel regions. This severely impacts the device performance. Contrastingly, in devices that incorporate 2D van der Waals (vdW) metal contacts, it is possible to maintain an ultraclean interface between the TMD material and metal.

## 4. Electron doping of graphene

Before we measure the Dirac-source diode, we measured doping level of graphene (Charge neutrality point, CNP). As shown in Supplementary Figure 5a, we measured CNP of graphene with two-probe measurement. By sweeping $V_{BG}$, CNP of graphene exhibits at $V_{BG}$=+1.9V, which indicates the Fermi level of graphene is located near the Dirac point.

## 5. Ohmic and non-Ohmic contact behaviors of graphene and graphite electrodes on $MoS_2$

Supplementary Figure 6 illustrates the contact behaviors of the graphene–$MoS_2$–graphene and graphite–$MoS_2$–graphite heterostructures. As reported previously[4], the graphene contact demonstrates a nearly linear I–V curve, which represents Ohmic behavior. In contrast, the graphite contact demonstrates a nonlinear I–V curve and non-Ohmic behavior

## 6. Theoretical backgrounds of DS diode

### 6. 1 Analytical formula for ideality factor less than one of DS diode

The Dirac point of graphene is above the conduction band edge of $MoS_2$ as show in Fig. 1d, and the doping of graphene is p-type at a negative gate voltage. When the bias voltage applied to graphene decreased, the injected current density increased differently from that in the case of normal metals. The current is described using Landauer approach, as follows:

$$\mathrm{I} = \frac{2q}{h}\int_{-\infty}^{\infty} dE T(E) M(E)[f(E - E_{F,S}) - f(E - E_{F,D})], \qquad (1)$$

where T(E) is the transmission at energy E, M(E) is the number of channels at energy E, f(E) is the Fermi distribution function of the contact, and $E_{F,S}$ and $E_{F,D}$ are the Fermi levels of the source and drain, respectively. In this case, $E_{F,S} = 0$, $E_{F,D} = E_{F,S} - qV_{bias} = -qV_{bias}$. Because graphene has a linear density of state, $\mathrm{M(E)} = M_0|E - E_D|$, where $E_D$ is the Dirac point of the graphene source. The thermionic current has a transmission, $\mathrm{T(E)} = 1$. Applying the above conditions to Equation 1, we obtain:

$$\mathrm{I} = \frac{2qM_0}{h}\int_{-\infty}^{\infty} dE|E - E_{Dirac}|[f(E) - F(E + qV_{bias})]. \qquad (2)$$

When the Dirac point of graphene is above the top of barrier of $MoS_2$, the energy between the Dirac point of graphene and the top of barrier of $MoS_2$ is larger than a few $k_BT$.

$$\mathrm{I} = \frac{2qM_0}{h}\left(1 - e^{-\frac{qV_{bias}}{k_BT}}\right)\left\{\int_{E_{top}}^{E_D} dE(E_D - E)e^{-\frac{E}{k_BT}} + \int_{E_D}^{\infty} dE(E - E_D)e^{-\frac{E}{k_BT}}\right\}$$

$$= \frac{2qM_0k_BT}{h}\left(1 - e^{-\frac{qV_{bias}}{k_BT}}\right)\left[2k_BTe^{-\frac{E_D}{k_BT}} - (E_{top} + k_BT - E_D)e^{-\frac{E_c}{k_BT}}\right]$$

$$\sim \frac{2qM_0k_BT}{h}\left(1 - e^{-\frac{qV_{bias}}{k_BT}}\right)[E_D - E_{top} - k_BT]e^{-\frac{E_c}{k_BT}}$$

$$= J_0\left(1 - e^{-\frac{qV_{bias}}{k_BT}}\right)[E_D - E_{top} - k_BT], \qquad (3)$$

where $E_{top}$ is the top of barrier of $MoS_2$.

From Equation 4, η can be defined as

$$\eta = -\frac{q\partial V_{bias}}{k_B T \partial log I_D} = \frac{1}{1 + \frac{k_B T}{E_D - E_{top} - k_B T}} \tag{4}$$

and when $E_D - E_{top} = nk_B T$,

$$= \frac{1}{1 + \frac{1}{n-1}} = \frac{n-1}{n} < 1, \tag{5}$$

i.e., η becomes less than 1.

### 6. 2 Theoretical calculations and simulations of DS diode

Quantum transport simulations are performed to explore switching properties of DS Schottky diode. The DS diode consists of graphene, monolayer (ML) $MoS_2$ and graphite vdW heterojunction with graphene as a cold electron source. Both graphene and ML $MoS_2$ are described by a Dirac Hamiltonian[5]:

$$\widehat{H}_0 = at(\tau k_x \widehat{\sigma}_x + k_y \widehat{\sigma}_y) + \frac{\Delta}{2}\widehat{\sigma}_z \tag{6}$$

Where, a is the lattice constant, t is the intralayer hopping energy and $\Delta$ is the band gap which is 0 eV for graphene and 1.65 eV for ML $MoS_2$ [5]. $\widehat{\sigma}$ denotes Pauli metrics on the Pseudo spin basis. The van der Waals coupling parameters between graphene and ML $MoS_2$ are obtained by fitting the band structure of graphene-$MoS_2$ heterojunction calculated by using density functional theory (DFT). Graphite is modeled by n-type bilayer graphene Hamiltonian to simplify calculations[7]. The ballistic transport properties of DS diode are calculated by solving the Schrodinger equation within the non-equilibrium Green's function (NEGF) and Poisson's equation self-consistently[5].

Supplementary Figure 7 shows the device structure and transport properties of the simulated DS diode. The device consists of 10 nm graphite, 32 nm $MoS_2$ and 8 nm graphene. An Ohmic contact is used between p-type graphene and 7 nm n-type $MoS_2$ as shown in Supplementary Figure 1a. The Schottky barrier between graphite and $MoS_2$ is set at 0.48 eV. The contact types are consistent with the fabricated DS diode. The top gate length is 20 nm and bias voltage is applied on p-type graphene. $MoS_2$ under the gate has a flat band at $V_G$= 0 V. It is shown that a steep slope switching is realized in DS diode at $V_G$ = 0.1 V in Supplementary Figure 7b and the ideality factor can be as small as 0.69 at -0.15 V < $V_{bias}$ < -0.10 V, which breaks the switching

limit of conventional Schottky diode. At $V_{bias}$ = -0.05 V, the Dirac point of graphene is below the top of barrier of $MoS_2$ as shown in Supplementary Figure 7c. As bias voltage is decreased to -0.15 V, the Dirac point of graphene gets larger than the Schottky barrier at graphite-$MoS_2$ interface as shown in Supplementary Figure 7d. The injected carrier around the Dirac point is effectively filtered as calculated current density in Supplementary Figure 7d. Due to the linear density of states of graphene, the current is increased super-exponentially and ideality factor is less than 1 as expected. The current is increased by over 5 orders of magnitude with the average ideality factor about 0.88 as bias voltage is decreased from -0.10 V to -0.40 V. Besides promising steep slope switching, DS diode also have high on-state current over $10^3$ μA/μm and large rectifying ratio over $10^7$.

We also studied the impact of the doping level of graphene on switching properties of DS diode as shown in Supplementary Figure 7b. The ideality factor of DS diode with p-type graphene is less than one at the bias voltage region between -0.1 V and -0.3 V, and the current is increased over four orders of magnitude. While, ideality factor gets larger than one as graphene is intrinsic or n-type as shown in Supplementary Figure 8a, because the Dirac point is always below the top of channel barrier and cannot filter high energy thermionic current. Supplementary Figure 8b shows that there is an obvious phase transition of ideality factor from sub-unity to over-unity as graphene is doped from p-type to n-type.

Next, we studied electron doped region of graphene to realize sub-unit ideality factor. There are two important factors to realize ideality factor less than one in DS diode: the doping type of graphene and the Schottky barrier height between graphite and ML $MoS_2$. In order to realize sub-unity ideality factor in electron doped region of graphene, a negative gate voltage has to be applied to achieve carrier transport by valence band of ML $MoS_2$. We first fix the Schottky barrier height ($\Phi_B$ = 0.48 eV) between graphite and ML $MoS_2$ as that in hole doped region of graphene, and apply n-type graphene with p-type Ohmic contact between graphene and ML $MoS_2$. Supplementary Figure 9a, b show such device cannot reach ideality factor less than one when current is larger than $1\times10^{-10}$ μA/μm, because there is a larger p-type Schottky barrier height between graphite and ML $MoS_2$. The current is mainly tunneling current through the p-type Schottky barrier and the Dirac point of graphene is not decisive to the transport as shown in Supplementary Figure 9c. If the Schottky barrier height is set at $\Phi_B$ = 1.17 eV, sub-unity ideality factor can be obtained in DS diode with electron doped graphene as

shown in Supplementary Figure 9a, b. At $V_{bias}$ = 0.15 V, the Dirac point of graphene gets lower than the top of channel barrier and can filter thermionic carriers over the barrier for sub-unity switching as shown in Supplementary Figure 9d.

### 7. Rectifying mechanism of metal/n-semiconductor Schottky diodes.

Supplementary Figure 10 shows the band diagram of metal/n-semiconductor junction at equilibrium, reverse bias regime, and forward bias regime. When metal/n-semiconductor junction is formed, Schottky barrier is formed due to the difference between the work function of metal ($\Phi_M$) and the electron affinity of n-type semiconductor ($\chi_{SC}$). In this junction case, Schottky barrier height (SBH, $\Phi_B$) is given by; $\Phi_B = \Phi_M - \chi_{SC}$. In reverse bias regime (negative bias on metal side or positive bias on semiconductor side), due to the SBH, only small leakage current can flow from metal to semiconductor. Since the forward bias condition is satisfied when we apply negative bias to graphene with graphite contact grounded, the Schottky barrier is formed at the interface of graphite/$MoS_2$, not graphene/$MoS_2$.

### 8. Schottky barrier height measurement

The Schottky barrier height can be altered by modifying the applied gate voltage. Supplementary Figure 11a depicts the measured Schottky barrier heights. Owing to limitations of the measurement system used in this study, measurements could only be performed in the voltage range of $V_{BG}$ = -12 V to 16 V. At the positive end of the applied $V_{BG}$ values, the device exhibits a linear I–V characteristic, thereby indicating a near-Ohmic contact. Accordingly, an increase in $V_{BG}$ causes a reduction in the graphite and graphene work functions[6]. This decreases the Schottky barrier heights of the graphene–$MoS_2$ and graphite–$MoS_2$ interfaces.

### 9. Dirac-source FET operation

Supplementary Figure 12 depicts the Dirac-source FET operation in our DS-diode. As shown in Fig. S12a, when the negative $V_{BG}$ is applied and swept control-gate voltage, injected hole carriers from the graphene is super-exponentially increases. Supplementary Figure 12b shows the characteristic $I_D$ versus control-gate ($V_{CG}$) transfer curve in cold carrier injection regime. Our DS diode operates as a Dirac-source FET, which exhibits the $SS_{ave\_1dec}$ and $SS_{ave\_3dec}$ 53.6mV/dec and 58.75mV/dec, respectively.

## 10. Thickness characterization of $MoS_2$ and graphene via Raman spectra analysis

Supplementary Figure 14 depicts the Raman spectra of $MoS_2$ and graphene used in the DS diode. As can be seen, the $MoS_2$ spectra are characterized by the $E^1_{2g}$ (in-plane) and $A_{1g}$ (out-of-plane) peaks. In this study, the thickness characterization of $MoS_2$ was performed by comparing the peak-to-peak distance between the $E^1_{2g}$ and $A_{1g}$ modes[7]. In the $MoS_2$ case, this distance equaled approximately 18.8 $cm^{-1}$, thereby indicating the use of an $MoS_2$ monolayer in the device. Meanwhile, the Raman spectra of graphene are characterized by the *G* and *2D* peaks, and the graphene-layer thickness can be determined by evaluating the intensity ratio of the *G* and *2D* modes, i.e., $I_{2D}/I_G$[8]. As observed, the graphene monolayer yields an $I_{2D}/I_G$ value that exceeds unity. This result is consistent with $I_{2D}/I_G$ value determined using the Raman spectra of graphene, i.e., $I_{2D}/I_G \approx 3$.

## 11. Temperature dependent measurement of DS diode

Supplementary Figure 15a shows Temperature dependent DS-diode measurement from 300K to 350K at fixed gate voltages $V_{CG}$=0V, $V_{TG}$=-0.7V, and $V_{BG}$=-6V. We observe that averaged ideality factor decreases with temperature in Supplementary Figure 15b. The ideality factor of DS diode is simplified as follows according to Eq. (4)：

$$\eta = 1 - \frac{k_B T}{E_D - E_{top}} \qquad (7)$$

Where $E_D - E_{top} > nk_bT$. The energy difference between $E_D$ **and** $E_{top}$ does not change and the ideality factor gets smaller as the increasing of temperature as shown in Supplementary Figure 15b**.** The measured temperature dependence of ideality factor can be well described by the formula (the red solid line) as shown in Supplementary Figure 15b. It should be noted that ideality factors can only be compared at the same temperature: the smaller the ideality factor, the better switching performance. The decreasing of ideality factor as a function of temperature does not mean the improvement of switching properties.

## 12. Repeatability test of DS diode

To ensure the repeatability of our DS diode, we performed 10 consecutive I-V measurement and found that our device shows the same I-V curve with the same ideality factor of $\eta_{ave\_1dec}$=0.84.

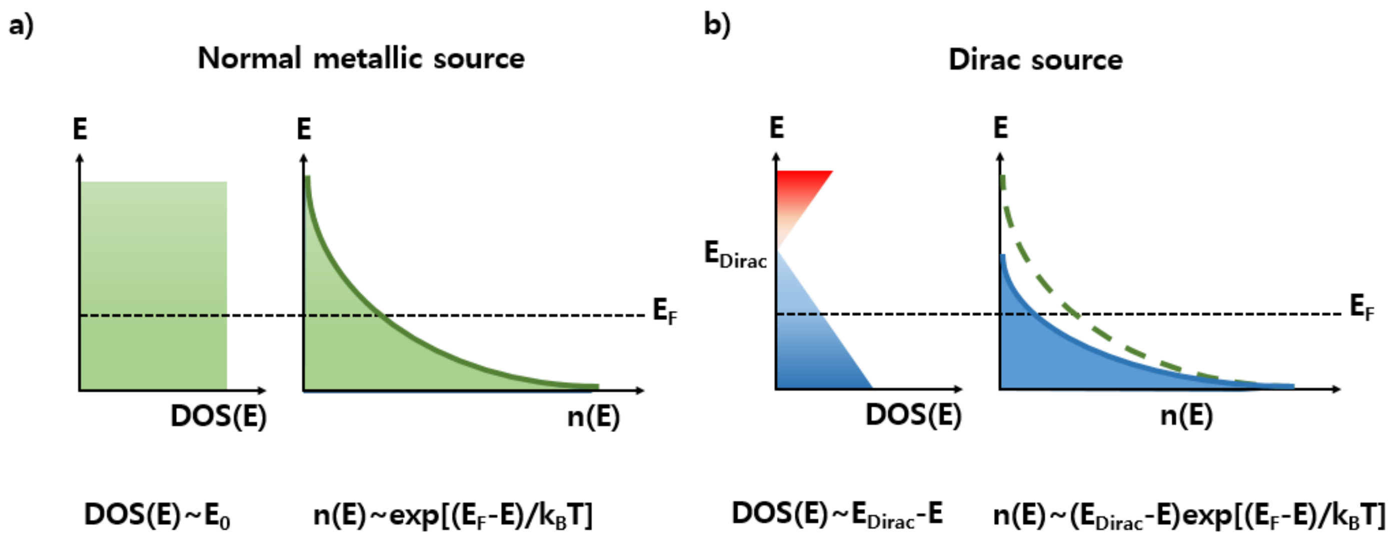


**Supplementary Figure 1. State and electron densities of normal metallic and Dirac sources.** (a) State and electron density of normal metallic source. In normal metallic source case, electron density follows Boltzmann distribution. (b) State and electron density of Dirac source. Dirac source shows super-exponentially decaying electron density. Green dashed line indicates Boltzmann distribution of normal metallic case.

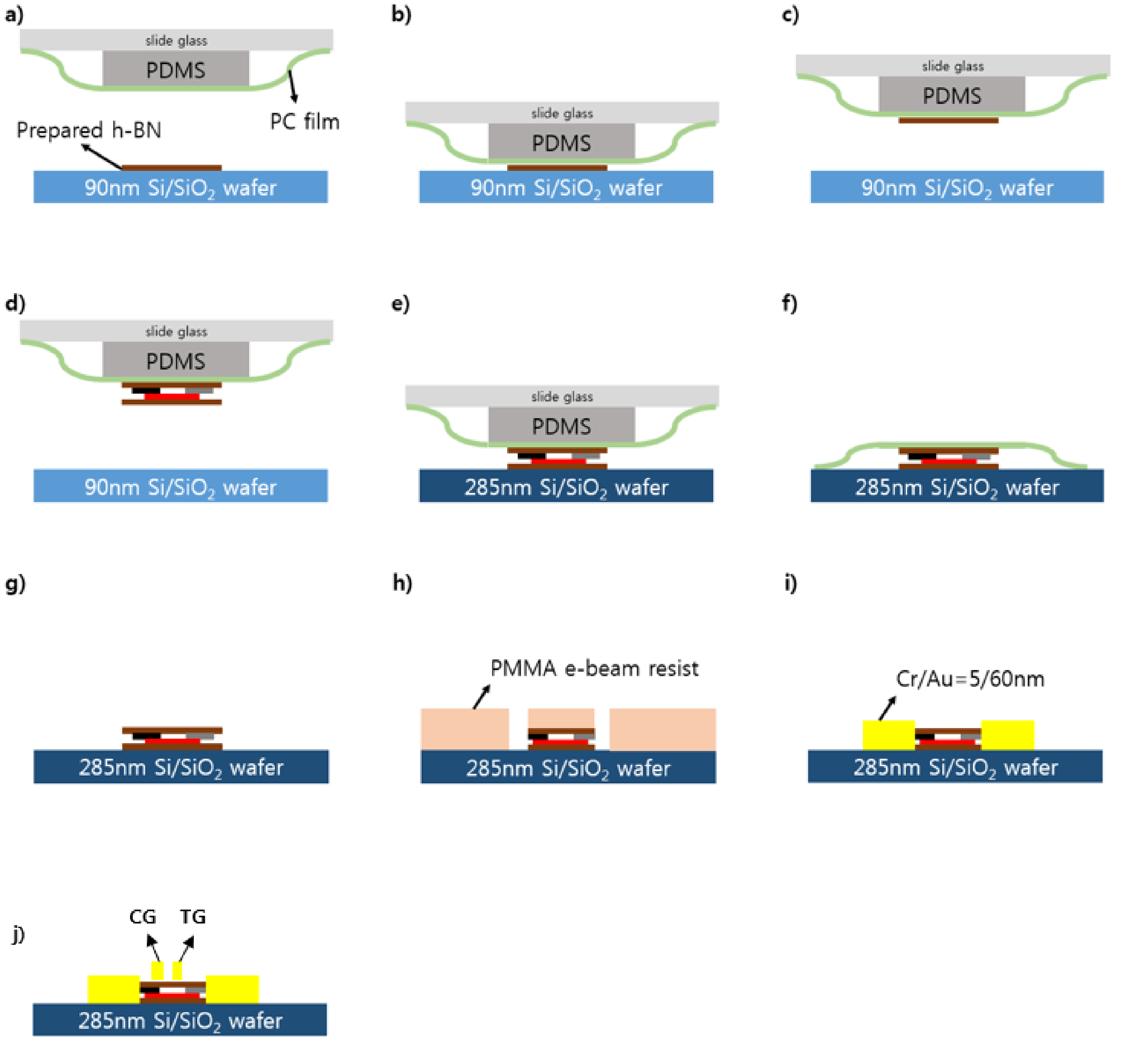


**Figure. S2 Fabrication of graphene–$MoS_2$–graphite heterojunction device.** (a) preparation of PDMS stamp covered with PC film on slide glass and mechanically exfoliated flakes (h-BN, graphene, graphite, and $MoS_2$) on 90-nm Si/$SiO_2$ wafer, (b, c) flake pick-up using PC film, (d) using the method described in (b) and (c), pick up of graphene, graphite, $MoS_2$, and bottom h-BN in that order for heterostructure fabrication, (e, f) placement of stacked heterojunctions on the prepared 285-nm Si/$SiO_2$, (g) washing of PC film, (h) E-beam lithography and etching, and (i) evaporation of Cr/Au layer (5/60-nm-thick) and lift-off. (j) Additional e-beam lithography and evaporation (Cr/Au) were performed to put top- and control-gate electrodes.

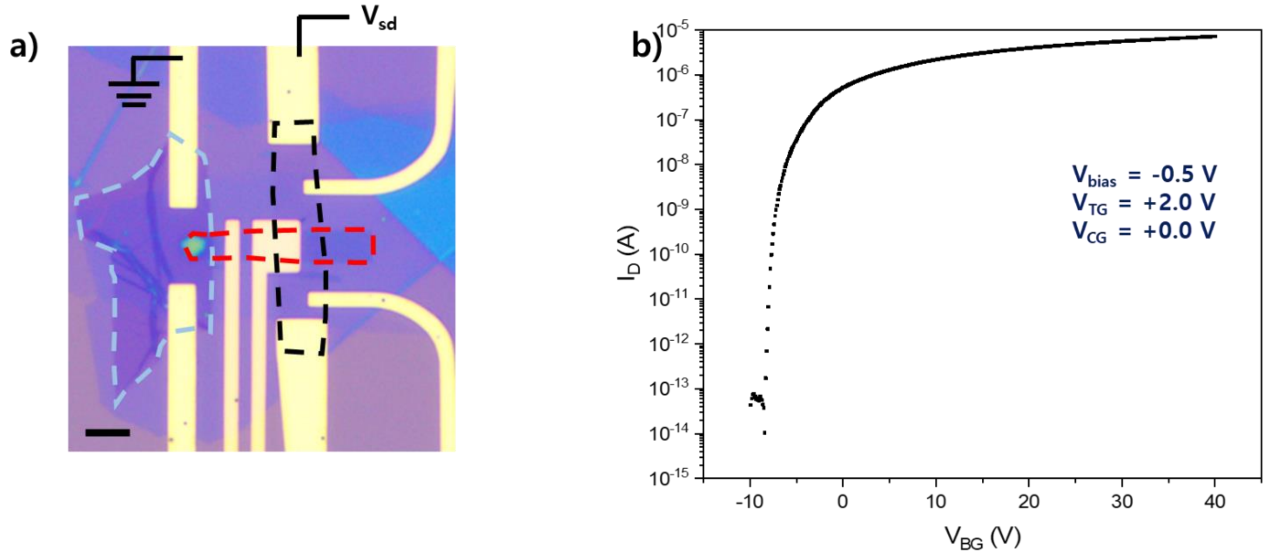


**Figure. S3 Transfer characteristic of DS-diode.** (a) Optical image of DS-diode. Grey, red, and black dashed lines indicate graphite, monolayer $MoS_2$, and graphene, respectively. Scale bar 5um. (b) Two-probe measurement of $MoS_2$ channel. At zero gate voltage, MoS2 channel showed n-type transfer characteristic.

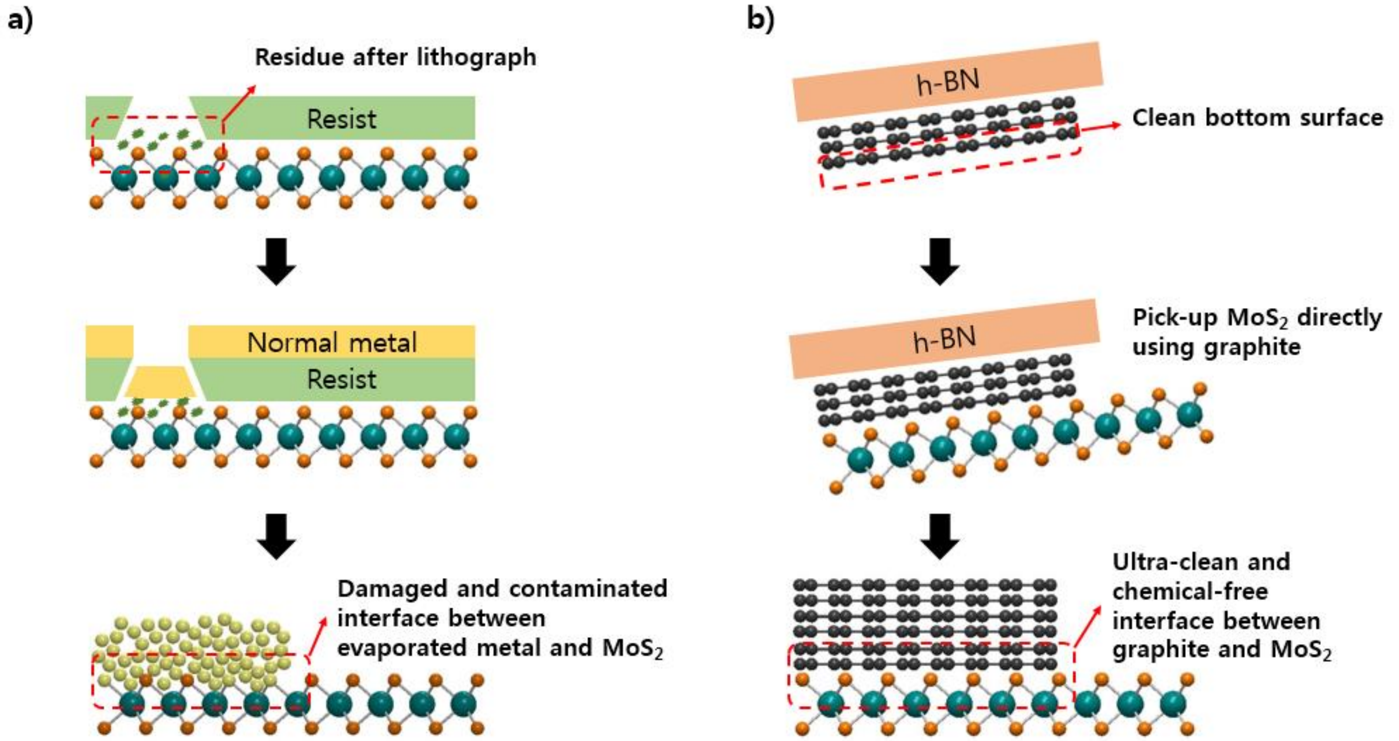


**Figure. S4 Comparison between direct metal deposition and 2D metal contacts.** (a) standard lithography and metal evaporation process and (b) 2D vdW metal contact process.

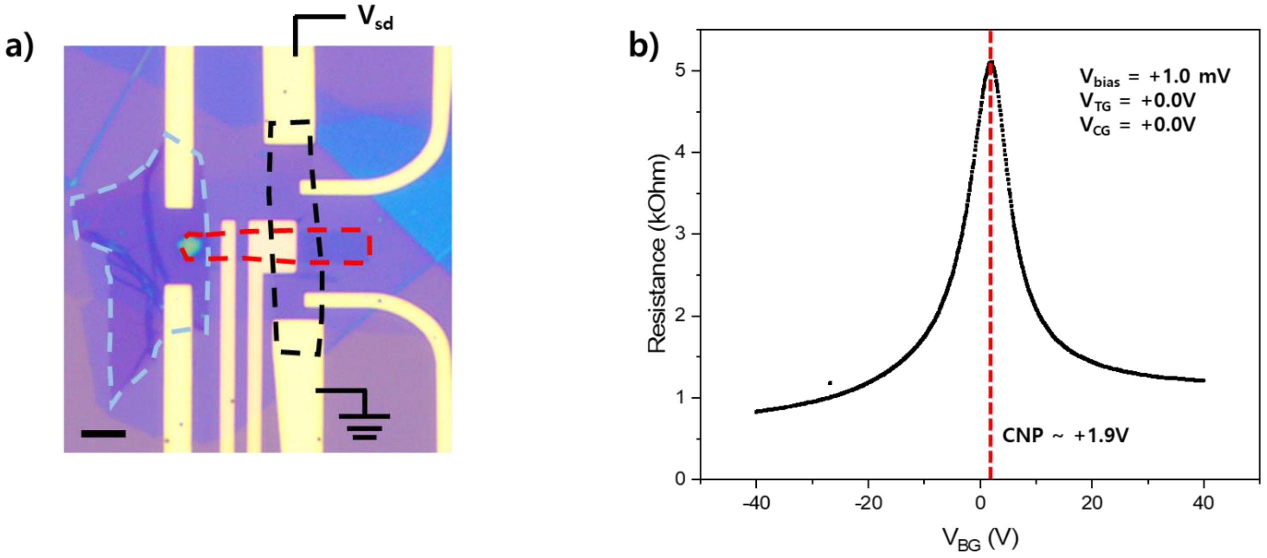


**Figure. S5 Charge neutrality point (CNP) of graphene.** (a) Optical image of DS-diode. Grey, red, and black dashed lines indicate graphite, monolayer $MoS_2$, and graphene, respectively. Scale bar 5um. (b) Two-probe measurement of graphene. Graphene exhibits CNP at $V_{BG}$=+1.9V, which indicates the Fermi level of graphene is located near the Dirac point.

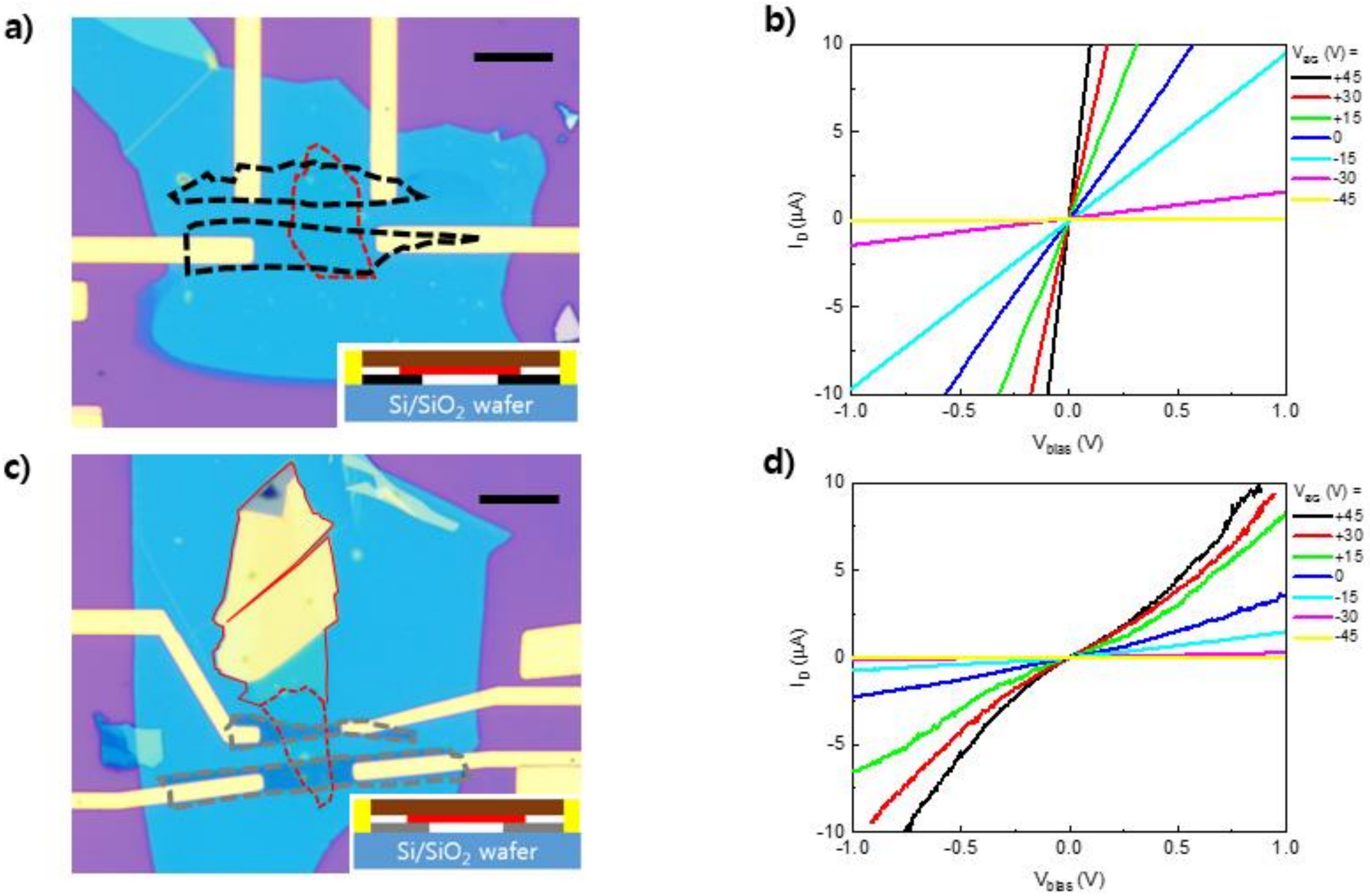


**Figure. S6 Ohmic and non-Ohmic contact behaviors of graphene and graphite electrodes on $MoS_2$.** Optical images of (a) graphene–$MoS_2$–graphene and (c) graphite–$MoS_2$–graphite interfaces along with corresponding transfer curves—(b) and (d), respectively. In (a) and (c), the black, gray, brown, red, and yellow colors represent graphene, graphite, h-BN, $MoS_2$, and contact metal (Cr/Au), respectively. Scale bar, 10μm.

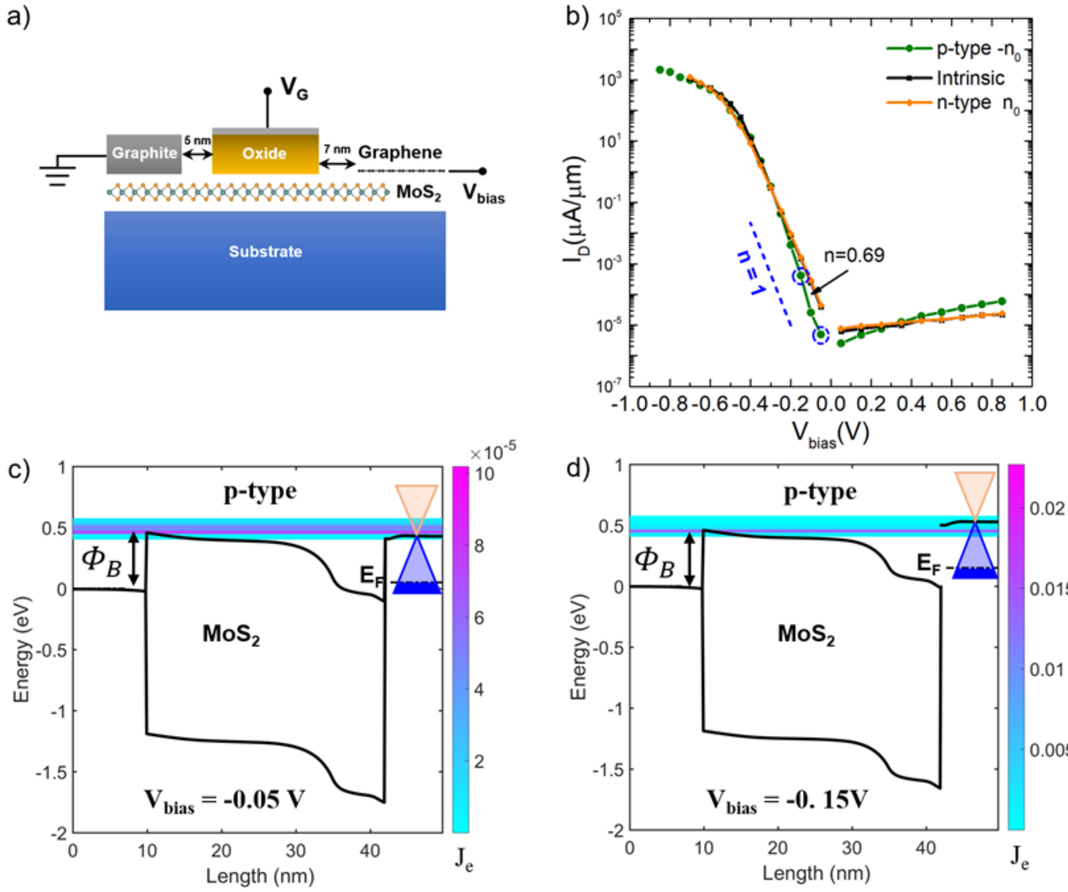


**Figure. S7 Device structure and transport properties of the simulated DS diode.** (a) Schematic image of the simulated graphene–$MoS_2$ diode with 10 nm graphite, 32 nm $MoS_2$ and 8 nm graphene. Graphite-$MoS_2$ and graphene-MoS2 contacts are Schottky type and Ohmic type respectively, which is consistent with the experiment. (b) $I_D$ -$V_{bias}$ curve of the simulated DS diode. Band diagram and current density of DS diode at (c) $V_{bias}$ = -0.05 V and (d) $V_{bias}$ = -0.15 V. $\boldsymbol{\Phi_B}$ is the Schottky barrier between graphite and $MoS_2$. $E_F$ is the Fermi level of graphene and the Fermi level of graphite is set at 0 eV. Unit of color bar μA/μm·eV.

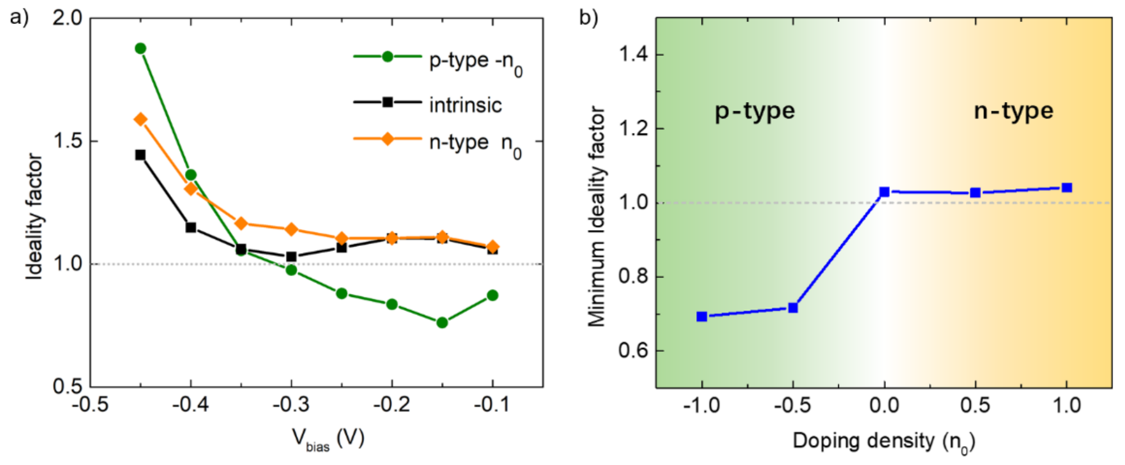


**Figure. S8 Ideality factor depend on the doping types of graphene.** (a) Ideality factor as a function of $V_{bias}$ of DS diode with different doping types of graphene. (b) The minimum ideality factor as a function of graphene doping density of DS diode.

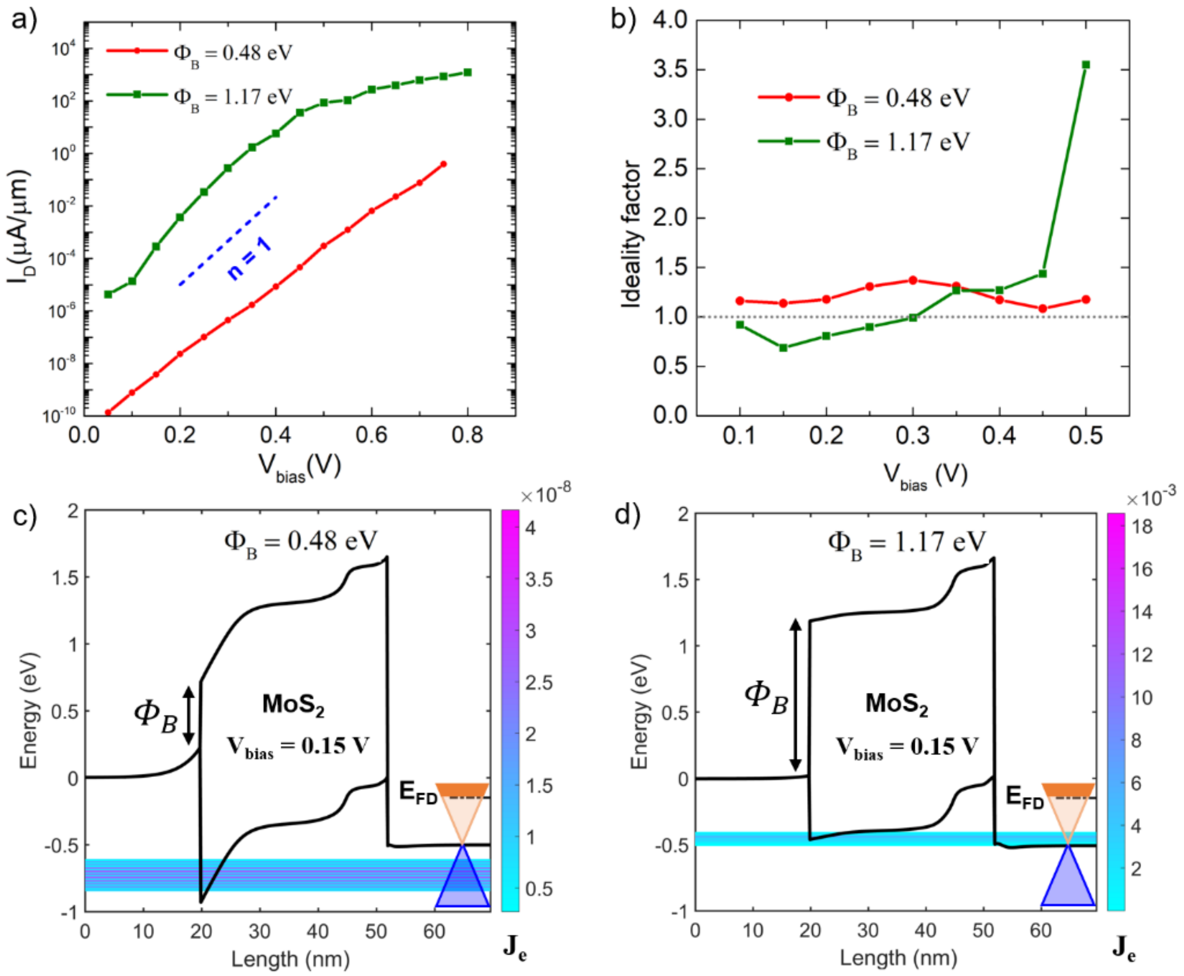


**Figure. S9 Simulated DS diode with p-type $MoS_2$.** (a) $I_D$ and (b) ideality factor as a function of $V_{bias}$ of DS diode with n-type doped graphene and different Schottky barriers between graphite and ML $MoS_2$. The contact between graphene and ML-$M_OS_2$ is assumed as p-type Ohmic contact. Band diagram and current density of

DS diode with different Schottky barriers between graphite and ML $MoS_2$ at $V_{bias}$ = 0.15 V: (c) $\Phi_B$ = 0.48 eV and (d) $\Phi_B$ = 1.17 eV. $E_{FD}$ is the Fermi level of graphene. Unit of color bar μA/μm·eV.

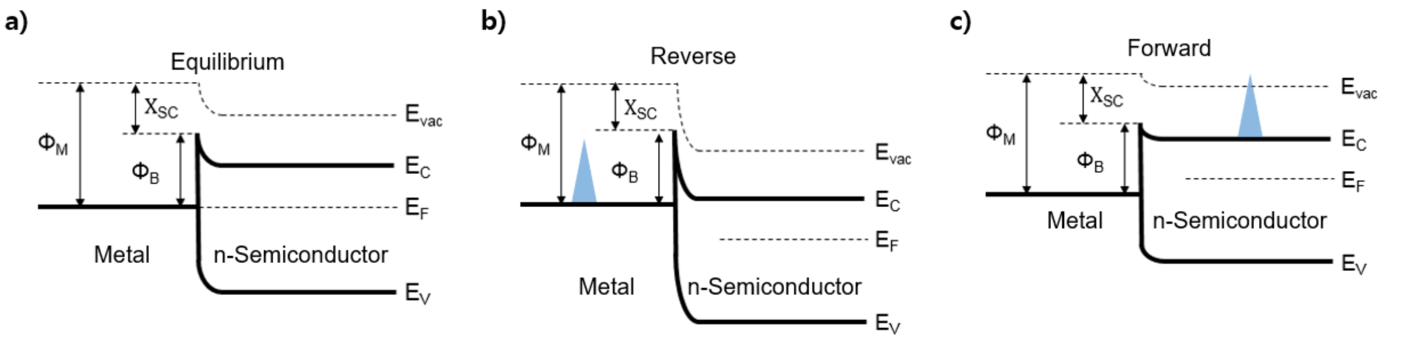


**Figure. S10 Band diagrams of metal/n-type semiconductor Schottky junctions. (a)** Band diagram at equilibrium state. Schottky barrier height ($\Phi_B$) is given by $\Phi_M$ -$\chi_{SC}$. **(b)** Band diagram at reverse bias regime (negative bias on metal side or positive bias on semiconductor side). Due to the Schottky barrier, only small leakage current from the metal can flow to the n-semiconductor. **(c)** Band diagram at forward bias regime (positive bias on metal side or negative bias on semiconductor side).

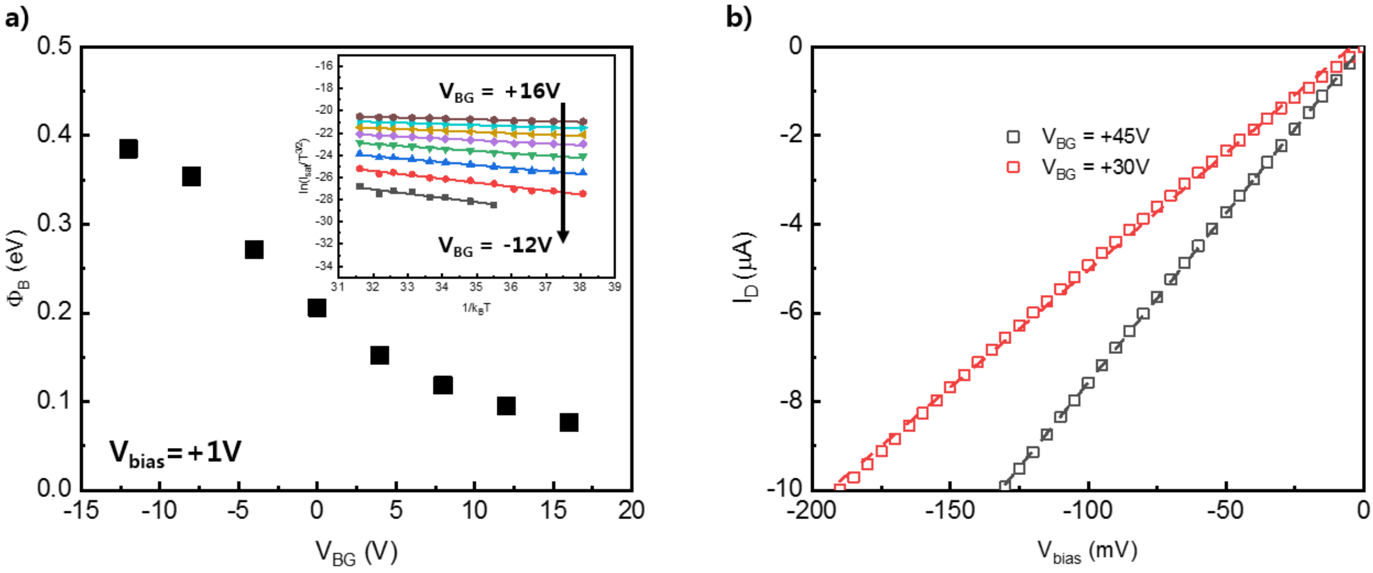


**Figure. S11 Schottky barrier height measurement.** (a) Arrhenius plot in reverse bias regime (inset) and Schottky barrier heights obtained in this study, (b) current versus bias voltage characteristic in the linear non-

diode regime.

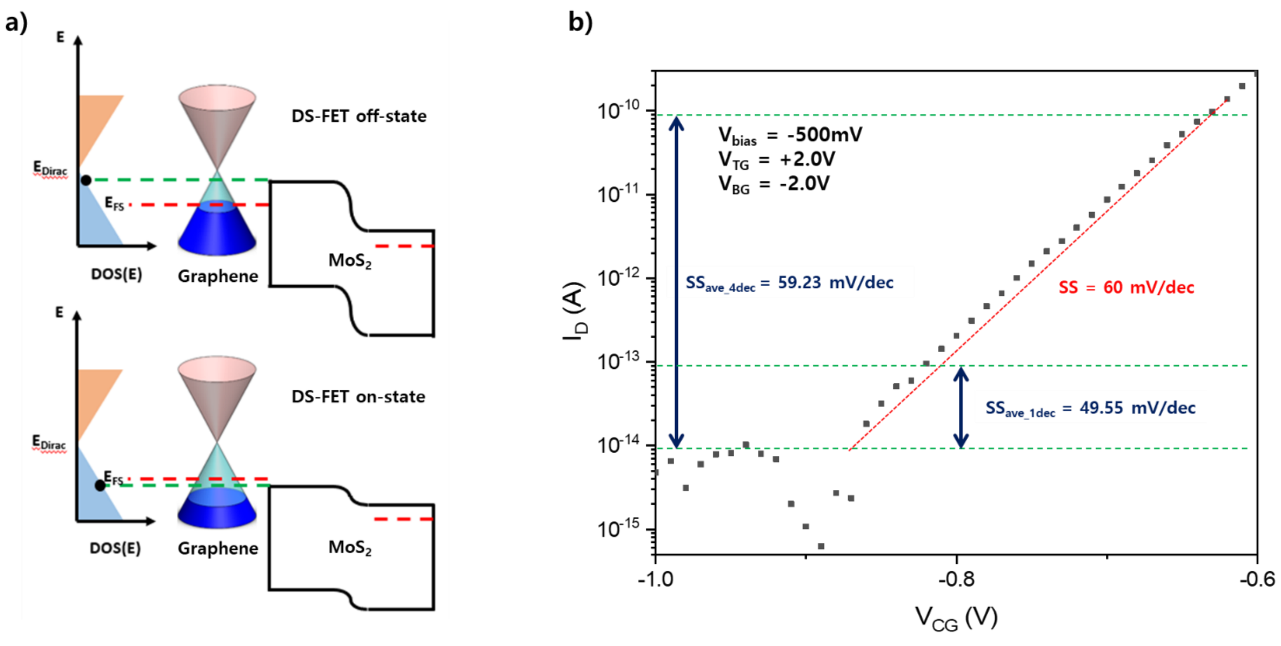


**Figure. S12 $MoS_2$ DS-FET measurement and its band diagram. (a)** Band diagrams of DS-FET. As the control-gate voltage, $V_{CG}$, is applied to the $MoS_2$ channel and graphene/$MoS_2$ overlapped region, the injection current density increases. **(b)** Transfer curves of the DS-FET at $V_{BG} = -3V$, $V_{TG}=+2V$, and $V_{sd} = -0.5$ V. The $SS_{ave\_1dec}$ and $SS_{ave\_4dec}$ exhibits 49.55mV/dec and 59.23mV/dec, respectively.

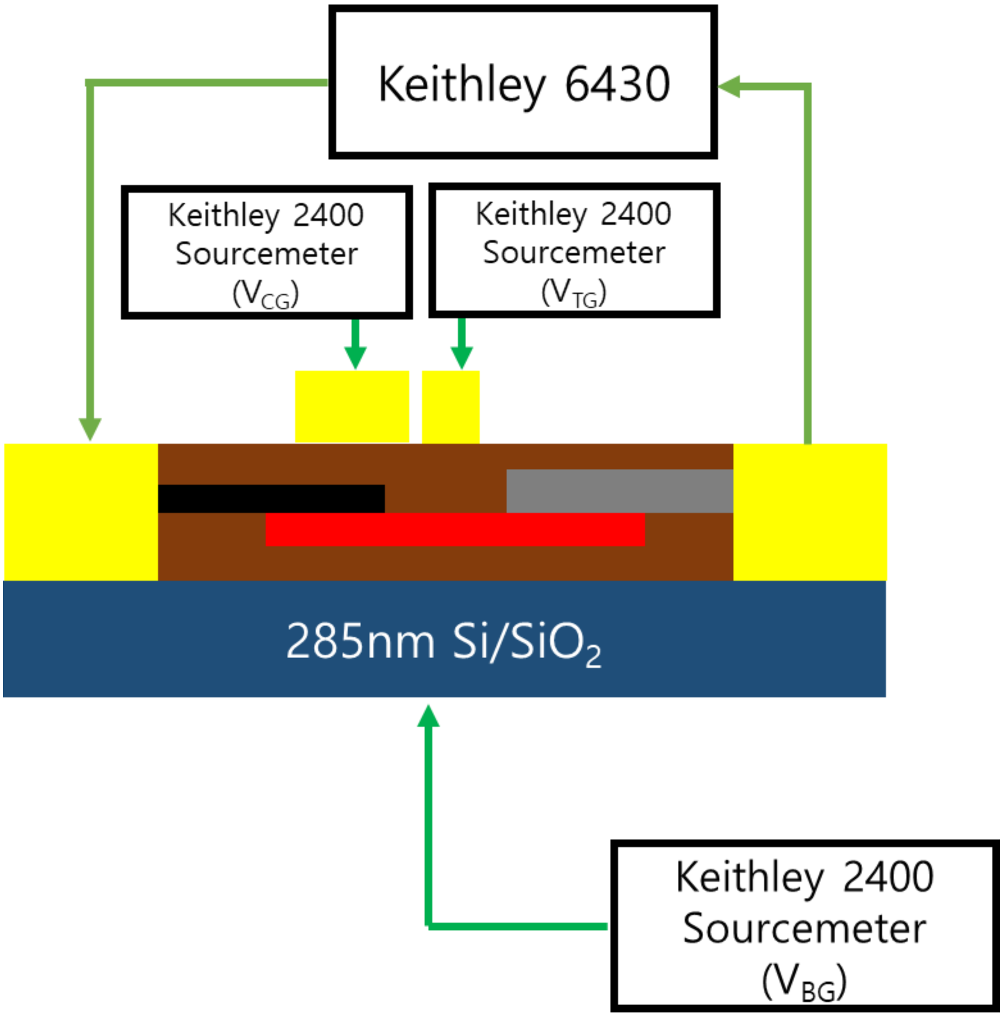


**Figure. S13 Measurement protocol.** Red, black, gray, brown, and yellow colors denote $MoS_2$, graphene, graphite, h-BN, and contact metal (Cr/Au = 5/60-nm-thick), respectively.

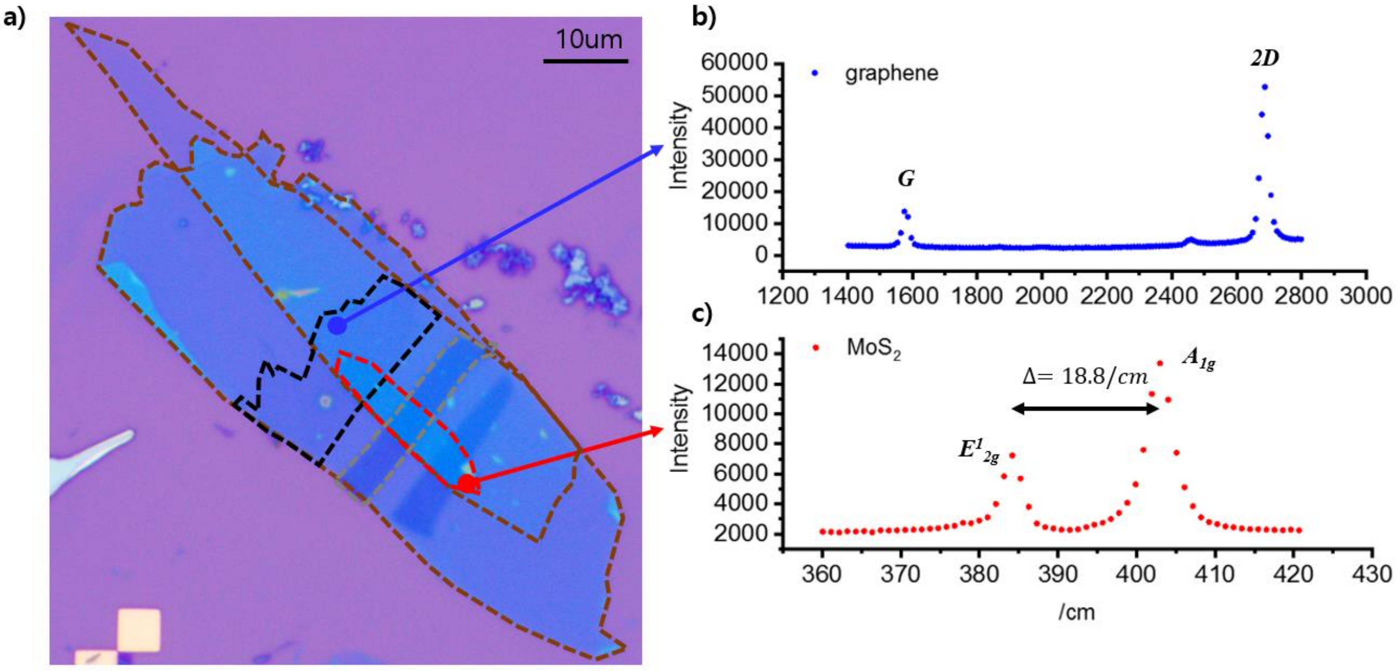


**Figure. S14 Raman spectra measurements of graphene and monolayer $MoS_2$. (a)** Optical image of graphene–$MoS_2$–graphite heterojunction device. The blue and red dots indicate the positions of the graphene and $MoS_2$ Raman spectra measurements, respectively. Scale bar, 10μm. **(b)** Raman spectra of graphene, and **(c)** Raman spectra of $MoS_2$.

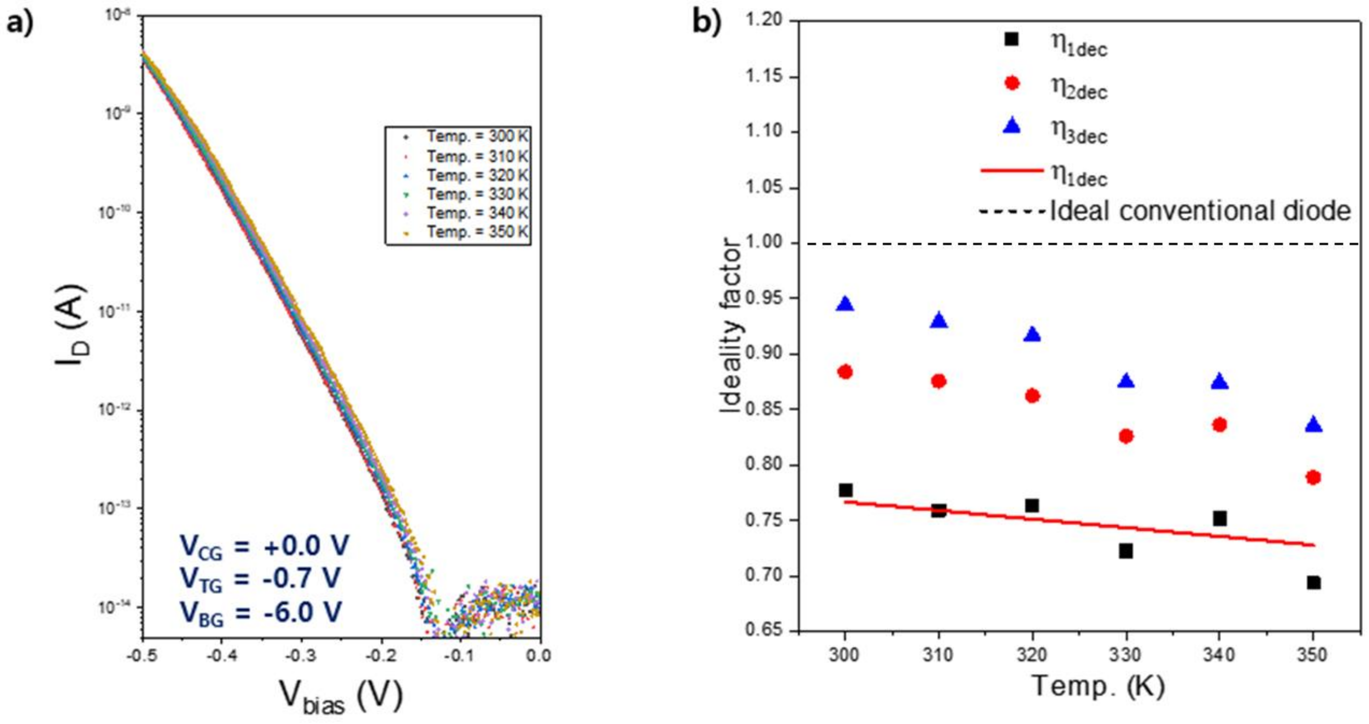


**Figure. S15 Temperature dependent DS-diode measurement. (a)** $I_D$ versus $V_{sd}$ characteristic transfer curves from 300K to 350K with fixed gate voltages $V_{CG}$=0V, $V_{TG}$=-0.7V, and $V_{BG}$=-6V **(b)** Temperature versus averaged ideality factor. The solid line is obtained by the analytic formula with $\boldsymbol{E_D - E_{top} = 0.11eV}$.

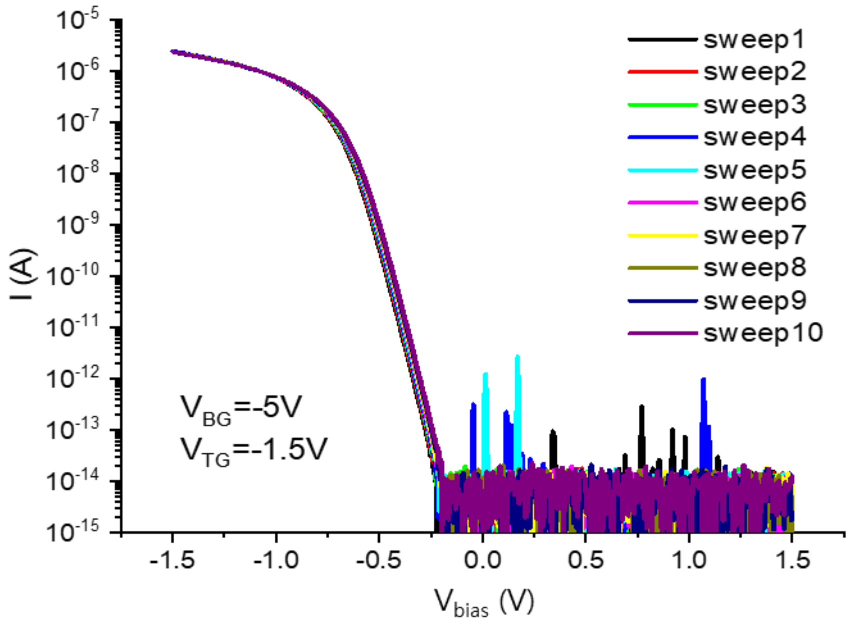


**Figure. S16 Repeatability test for DS diode.**

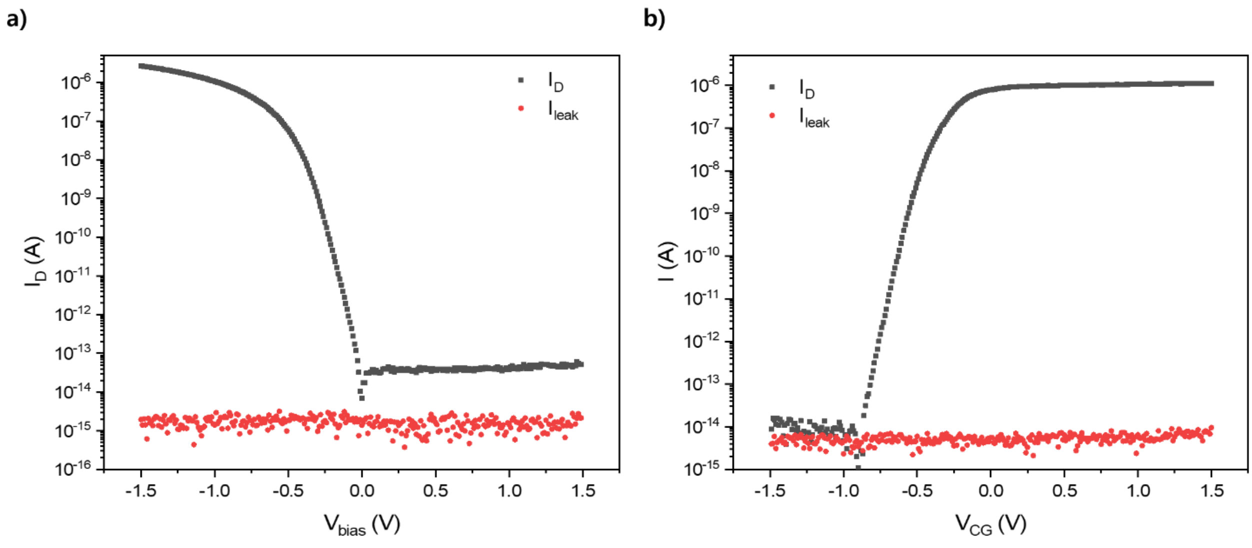


**Figure.S17 DS diode and DSFET with leakage current (a)** DS diode characteristic $I_D$-$V_D$ curve with system leakage current. Black and red dots denotes drain current and system leakage current, respectively. **(b)** DS FET characteristic $I_D$-$V_{CG}$ curve with control gate (CG) leakage current. Black and red dots denotes drain current and control gate (CG) leakage current.

## Supplementary references

1. Liu, Y. *et al.* Approaching the Schottky-Mott limit in van der Waals metal-semiconductor junctions. *Nature* **557**, 696-700 (2018). 10.1038/s41586-018-0129-8

2. Jung, Y. *et al.* Transferred via contacts as a platform for ideal two-dimensional transistors. *Nat. Electron.* **2**, 187-194 (2019). 10.1038/s41928-019-0245-y

3. Shen, P. C. *et al.* Ultralow contact resistance between semimetal and monolayer semiconductors. *Nature* **593**, 211-217 (2021). 10.1038/s41586-021-03472-9

4. Liu, F. *et al.* Dirac electrons at the source: breaking the 60-mV/decade switching limit. *IEEE Trans. Electron. Devices* 65, 2736-27743 (2018). 10.1109/TED.2018.2836387

5. Nilsson, J. *et al.* Electronic properties of bilayer and multilayer graphene. Phys. Rev. B 78, 045405 (2008). 10.1103/PhysRevB.78.045405

6. Liu, Y. *et al.* Toward barrier free contact to molybdenum disulfide using graphene electrodes. *Nano Lett.* **15**, 3030-3034 (2015). 10.1021/nl504957p

7. Liang, L. *et al.* First-principles Raman spectra of $MoS_2$, $WS_2$ and their heterostructures. *Nanoscale* **6**,5394-5401 (2014). 10.1039/C3NR06906K

8. Das, A. *et al.* Raman spectroscopy of graphene on different substrates and influence of defects. *Bull. Mater. Sci.* **31**, 579-584 (2008). 10.1007/s12034-008-0090-5
